# An Improved Hierarchical Genetic Algorithm for Sheet Cutting Scheduling with Process Constraints

**DOI:** 10.1155/2013/202683

**Published:** 2013-12-24

**Authors:** Yunqing Rao, Dezhong Qi, Jinling Li

**Affiliations:** ^1^The State Key Laboratory of Digital Manufacturing Equipment and Technology, Huazhong University of Science & Technology, Wuhan, Hubei 430074, China; ^2^Shenyang Donfon Titanium Industry Co., Ltd, Shenyang, Liaoning 110168, China

## Abstract

For the first time, an improved hierarchical genetic algorithm for sheet cutting problem which involves *n* cutting patterns for *m* non-identical parallel machines with process constraints has been proposed in the integrated cutting stock model. The objective of the cutting scheduling problem is minimizing the weighted completed time. A mathematical model for this problem is presented, an improved hierarchical genetic algorithm (ant colony—hierarchical genetic algorithm) is developed for better solution, and a hierarchical coding method is used based on the characteristics of the problem. Furthermore, to speed up convergence rates and resolve local convergence issues, a kind of adaptive crossover probability and mutation probability is used in this algorithm. The computational result and comparison prove that the presented approach is quite effective for the considered problem.

## 1. Introduction

The sheet cutting is widely used in engineering machinery, mining machinery, port machinery, and other industry machinery. The manufacturing model and management of modern enterprises have been changed greatly by the development of science and technology. Since integrated cutting stock has become a new cutting model, the integrated cutting stock brings much economic benefits to the enterprise, but it also brings some difficulty for solving the nesting and cutting scheduling problem in the meantime. Therefore, finding an advanced approach to solving the cutting scheduling problem for integrated cutting stock has an important practical and theoretical significance.

The processing capacity of different types of cutting machines is different. For example, 0.15 mm~6 mm thick steel plate can be cut on the laser cutting machine, and the cutting speed can reach 10000 mm/min. Meanwhile, 5 mm~200 mm thick steel plate can be cut on the flame cutting machine, and its cutting speed is 0–700 mm/min. Therefore, the cutting patterns of different material and thickness can be cut on the different types of cutting machines. In other words, there are some process constraints in the process of integrated cutting stock. Furthermore, the same cutting pattern can be cut on the different types of cutting machines with different cutting speed.

Assignment of *n* cutting patterns to *m* unit different cutting machines is considered as an integrated cutting scheduling problem. Each cutting pattern can be only cut one time on a machine. The objective of integrated cutting scheduling is minimizing the weighted completed time. It belongs to scheduling problem for nonidentical parallel machines with process constraint and sequence-independent setups.

Lots of algorithms and approaches are used in cutting stock problem including conventional optimization algorithms and various metaheuristic algorithms. For instance, Blazewicz et al. [1], Gomes and Oliveira[2], Umetani et al. [3], Bennell et al. [4], Cui et al. [5], Cui and Chen [6], Xie et al. [7], and others proposed some nesting algorithms and gave some suggestions on the two-dimensional cutting stock problem. However, literatures on the cutting scheduling problem (CSP) are not vast. There are some papers concerning the combined cutting stock and lot-sizing problem. NonÅs and Thorstenson [8] wanted to find an optimal production schedule involves the solution of a combined two-dimensional irregular cutting-stock and lot-sizing problem. In that paper, they proposed a problem formulation and suggested different solution algorithms (e.g., local search algorithms and simple tree-search algorithm) for a combined cutting-stock and lot-sizing problem. Gramani and França [9] analyzed the trade-off that arises when they solve the cutting stock problem by taking into account the production planning for various periods. In that paper, they formulated a mixed-integer mathematical model that combines the cutting stock and lot-sizing problems and developed a solution method based on an analogy with the network shortest path problem. NonÅs and Thorstenson [8] suggested a new column generating solution procedure (CGSP) for a combined cutting-stock and lot-sizing problem. Those papers are mainly concerned how to generate the cutting patterns, and minimize the trim-lost or trim-lost, setup cost and holding cost. However, how to assign those cutting patterns to different cutting machines, which belongs to cutting scheduling problem, is not concerned. As a good cutting scheduling can reduce production costs and raise the production efficiency, this problem is also important in the whole sheet cutting process. In this paper, our goal is to find a suitable cutting schedule and minimize the weighted completed time.

The cutting scheduling problem belongs to scheduling problem for nonidentical parallel machines with process constraint and sequence-independent setups. Scheduling problem has also been studied extensively, for example, Allahverdi et al. classified scheduling problems into batch and non-batch, sequence-independent and sequence-dependent setup, and categorizes the literature according to the shop environments of single machine, parallel machines, flowshops, and job shops [10]. Each scheduling problem is denoted by the standard threefield notation *α*/*β*/*γ*. The first field *α* describes the scheduling type, the second field *β* is reserved for the information and conditions of scheduling, while the third field *γ* contains the performance criteria. Cheng and Sin surveyed the major research results in deterministic parallel-machine scheduling [11]. And lots of the literatures of scheduling have considered parallel-machine scheduling problems. Peng and Liu, Anglani et al., Fowler et al., and other scholars have done some works on parallel machines problem [12–14]. And the vast majority of these studies have concentrated on studying the case of identical parallel machines. However, the case of nonidentical parallel-machine schedules has more practical sense than the case of identical parallel machines in real production (e.g. cutting and scheduling problem belong to nonidentical parallel-machine schedule).

Literatures on nonidentical parallel-machine schedules problem are not vast. Li and Yang gave a review of the nonidentical parallel-machine total weighted/weighted completion time problems [15]. Van Hop and Nagarur proposed a new approach to solve the PCB scheduling problem on a set of nonidentical machines. This approach model which related tasks of grouping, sequencing, and component switching as one integrated problem, with an objective of minimizing the total makespan [16]. Balin proposed a GA approach, which minimized maximum completion time (makespan) and considered nonidentical parallel machine scheduling problem with fuzzy processing times [17]. In order to adapt GA to nonidentical parallel machine scheduling problem, he proposed a new crossover operator and a new optimality criterion. Alcan and Balişgil presented a kind of genetic algorithm based on machine code for minimizing the processing times in nonidentical machine scheduling problem [18]. Also triangular fuzzy processing times were used in order to adapt the GA to nonidentical parallel machine scheduling problem in that paper. Besides the above mentioned papers, there are a few others that have investigated the unrelated-parallel machine scheduling problems with different approach (Liaw et al. [19], Rocha et al. [20], Mehravaran and Logendran [21], Arnaout et al. [22], Charalambous and Fleszar [23]). However, even though there are some papers considered nonidentical parallel-machine scheduling, few of them deal with nonidentical parallel machine with process constraints.

Taken together, how to assign cutting patterns to different cutting machines (i.e., the sheet cutting scheduling problem) is a key point for the whole sheet cutting process, but this issue are not involved in the previous papers. Considering the practical requirement, some further work on the sheet cutting problem needs to be done. To solving this problem, a mathematical model which takes into account the weighted completed time is presented. Furthermore, an improved hierarchical genetic algorithm (ant colony—hierarchical genetic algorithm) is developed to solve this mathematical model. Based on the characteristics of the problem, a hierarchical coding method is used in this algorithm. In addition, to speed up convergence rates and resolve local convergence issues, a kind of adaptive crossover probability and mutation probability is used in this algorithm.

The rest of the paper is organized as follows. In Section 2, a detailed description of the sheet cutting scheduling problem is illustrated. A mathematic model for the sheet cutting problem is presented in Section 3. In Section 4, a process for solving the sheet cutting problem with a hierarchical genetic algorithm is demonstrated. Then the results of computational experiment are described in Section 5. Finally, conclusions and directions are given in Section 6.

## 2. Statement of the Problem

**Figure 1:**
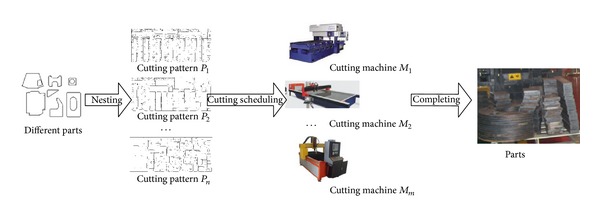
The production process of sheet cutting.

The process of sheet cutting is as follows. First, a variety of cutting patterns with the combination of different parts are generated by special nesting software. Second, those cutting patterns are assigned to different cutting machines. Thirdly, those cutting patterns are cut by different cutting machines. The process of sheet cutting can be showed by Figure 1. As the processing capacity of different types of cutting machines is different, a cutting pattern can be cut on some special cutting machines under different cutting speed. The capacity of some different cutting machines is shown in Table 1.

For example, a cutting pattern, material Q235 and thickness 20 mm, can be cut on plasma cutting machine B, flame cutting machine A, flame cutting machine B and flame cutting machine C. The cutting speed is 800 mm/min on the plasma cutting machine B, but it is 500 mm/min on the flame cutting machine C. Therefore, how to assign cutting patterns to different cutting machines is needed to be solved by some method.

## 3. Mathematical Modeling

We consider the cutting scheduling problem of *n* cutting patterns for *m* nonidentical parallel machines with process constraints in detail, where *m* < *n*. *Tp*_*ij*_ represents the processing time of cutting patterns, where *i* = 1, 2, …, *n*, *j* = 1, 2, …, *m*. It means the processing time of cutting pattern *P*_*i*_ on the cutting machine *M*_*j*_. Each cutting pattern has a corresponding weight coefficient *ω*_*i*_. We need to make an optimal scheduling to minimize the weighted completion time.

The problem considered can be summarized by the following points.

1. *n* different cutting patterns *P*_1_, *P*_2_, …, *P*_*n*_ need to be cut.
2. Each cutting pattern includes some information such as number of parts, cutting length, number of punch, and so forth.
3. *m* different cutting machines *M*_1_, *M*_2_, …, *M*_*n*_ can be used. Each cutting machine can cut a variety of sheets of different material and thickness.
4. The process time of a cutting pattern on different cutting machine may be different.
5. The products pass rate is 100%, in other words, there is no reprocessing.
6. Once starting cutting, it is not allowed to interrupt.
7. Each cutting pattern is independent.
8. Only one working procedure (i.e., cutting procedure) is included in this sheet cutting process.
9. The cutting process includes special process constraint, which the cutting machine set C*P*_*i*_ means cutting patterns *P*_*i*_ can be used, C*P*_*i*_ ∈ *M*, C*P*_*i*_ ≠ Φ.
10. Setup time includes punch time, collection time (the time of collect parts after completing cut) and the time using to adjust steel on the cutting machine.
11. Empty travel time is ignored, in other words, the processing time is the actual cutting time.

*Definition.* Consider the following:

 
*n* is the number of cutting patterns,
 
*m* is the number of cutting machines,
 
*i* is cutting pattern index, *i* = 1,…, *n*,
 
*j* is cutting machine index, *j* = 1,…, *m*,
 
*pn*_*i*_ is number of parts in cutting pattern *P*_*i*_,
 
*ph*_*i*_ is number of punch in cutting pattern *P*_*i*_,
  (1) $$\begin{array}{c} tp_{ij}\{\begin{array}{cc} \text{cutting}\;\text{time}\;\text{of}\;\text{cutting}\;\text{pattern}\;i\;\mathrm{on}\;\text{machine}\;j & \\ \;\;\text{if}\;\text{cutting}\;\text{pattern}\;P_{i}\;\text{can}\;\text{be}\; & \\ \;\;\text{processed}\;\mathrm{on}\;\text{machine}\;j & \\ \infty \;\text{if}\;\text{cutting}\;\text{pattern}\;P_{i}\;\text{cannot}\;\text{be} & \\ \;\text{processed}\;\mathrm{on}\;\text{machine}\;j, & \end{array} \end{array}$$
 
*Vpm*_*ij*_ is cutting speed of cutting pattern *P*_*i*_ on cutting machine *M*_*j*_,
 
*stp*_*ij*_ is setup time of cutting pattern *P*_*i*_ on cutting machine *M*_*j*_,
 
*ω*_*i*_ is process weight of cutting pattern *P*_*i*_,
 
*c*_*ij*_ is completed time of cutting pattern *P*_*i*_ on machine *M*_*j*_.

*Decision Variables*. Consider the following:

(2) $$\begin{array}{c} z_{ij}\{\begin{array}{cc} 1\;\text{if}\;\text{cutting}\;\text{pattern}\;P_{i} & \\ \text{can}\;\text{be}\;\text{cut}\;\mathrm{on}\;\text{cutting}\;\text{machine}\;M_{j} & \\ 0\;\text{otherwise}. & \end{array} \end{array}$$

*Objective Function*. Consider the following:

(3) $$\begin{array}{c} f=\min\sum_{i=1}^{n}\sum_{j,j\in \text{C}P_{i}}^{}\omega_{i}c_{ij}z_{ij} \end{array}$$

subject to

(4) $$\begin{array}{c} \sum_{j,j\in \text{C}P_{i}}^{}z_{ij}=1,\;i=1,2,\ldots ,n \end{array}$$

(5) $$\begin{array}{c} \sum_{i=1}^{n}\sum_{j,j\in \text{C}P_{i}}^{}z_{ij}=n,\;i=1,2,\ldots n,\;\;j=1,2,\ldots m \end{array}$$

(6) $$\begin{array}{c} c_{ij}=\left(c_{xj}+tp_{ij}+stp_{ij}\right)\times z_{ij}, \end{array}$$

(7) $$\begin{array}{c} stp_{ij}=ph_{i}\times 1+pn_{i}\times 0.5+5, \end{array}$$

(8) $$\begin{array}{c} tp_{ik}\times Vmp_{ik}=t_{il}\times Vmp_{il}\;k,l=1,2,\ldots ,m, \end{array}$$

(9) $$\begin{array}{c} \text{C}P_{i}\in M,\;\text{C}P_{i}\notin \Phi . \end{array}$$

The minimizing weighted completed time can be got by objective function (3). Equation (4) considers a cutting pattern can be cut only on one cutting machine. All of cutting patterns are sure to be cut by (5). Equation (6) represents the completed time of cutting pattern *P*_*i*_ on machine *M*_*j*_. It consists of three components which involve *c*_*xj*_, *t*_*pj*_ and *stp*_*ij*_, where *c*_*xj*_ represents the completed time of cutting pattern *P*_*x*_ which is cut on the cutting machine *M*_*j*_ before cutting pattern *P*_*i*_. There are three components for (7). The first component represents punch time (punch time of each drilling is 1 minute). The second component represents collection time (the time of collect each part which a cutting pattern includes cut is 0.5 minute). The last component relates to the time using to adjust steel on the cutting machine. Processing time of a cutting pattern *P*_*i*_ on different machines can be expressed by (8). Formula (9) considers the cutting process contains a special process constraint. In other words, a cutting pattern is assigned which cutting machine depends on the sheet characteristics.

With the increase of the problem size, the solution of the problem becomes very complicated, or it is even impossible to be solved with conventional optimization methods. And it has been proved it is a NP-problem. In this paper, an ant colony—hierarchical genetic algorithm is considered to solve this problem. A set of optimized initial solution is generated by ant colony algorithm which has some merits, such as simple and universal, robustness. However, it is easy to fall into local optimum. Then, the hierarchical genetic algorithm which has strongly global search capability is used to further optimize the initial solution. Fast and efficient global optimizing can come true by using the ant colony—hierarchical genetic algorithm.

## 4. Ant Colony—Hierarchical Genetic Algorithm

A set of optimized initial solution is generated by using the ant colony algorithm for its simple and universal [24]. Then the solution is furthermore optimized by hierarchical genetic algorithm for its strongly global search capability. A hierarchical structure, which is consisted of parameter genes and control genes, is used by the hierarchical genetic algorithm [25]. The parameter genes are the lowest level, and control genes are in the higher levels of the parameter genes. The parameter genes are controlled by the control genes. In addition, to speed up convergence rates and resolve local convergence issues, a kind of adaptive crossover probability and mutation probability is used in this algorithm.

### 4.1. Code

If the binary encoding is used for solving the cutting scheduling problem, the chromosome will be very complex and difficult for the crossover operator and decoding. So, to overcome these difficulties, the natural number encoding couple with binary encoding is used.

**Figure 2:**
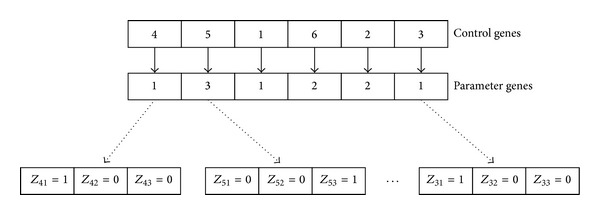
The process of natural number encoding.

For example, six cutting patterns are assigned to three different cutting machines. The cutting sequence is showed by control genes and the cutting machine of select is showed by parameter genes. The value of parameter gene is 1 means the selected cutting machine is *M*_1_. As is shown in Figure 2, the sequence of cutting patterns is *P*_4_, *P*_5_, *P*_1_, *P*_6_, *P*_2_, and *P*_3_ and the cutting pattern *P*_1_, *P*_2_, *P*_3_, *P*_4_, *P*_5_, and *P*_6_ are, respectively, assigned to *M*_1_, *M*_2_, *M*_1_, *M*_1_, *M*_3_, and *M*_2_. Each parameter gene can be further expressed by 0-1 variables *z*_*ij*_.

### 4.2. Initial Population

The initial population is generated by ant colony algorithm. The following gives the concrete solving process of ant colony algorithm.

*Selecting the Ants' Path*. It supposes that the ants movement between nodes which represent cutting patterns on different cutting machines and different amount of pheromone left on the nodes at the same time. Then the pheromone impacts the path of the next lot size ants moving [26]. *τ*_*z*_*ij*__(*t*) represents the pheromone values at *t*  (*t* = 0, 1, 2,…) moment on the different nodes. The initial of the pheromone value *τ*_*z*_*ij*__(0) = *ε* (the *ε* is a minimal number). There are *N*_ant_ ants distributed on the different nodes. Then each ant according to the pheromone value of next node and the heuristic factor independently chooses the next node. *p*_*z*_*ij*_*z*_(*i*+1)*x*__^*k*^(*t*) represents that the transition probability of ant *k*  (*k* = 1, 2,…, *N*_ant_) moves from the node *z*_*ij*_ to the next node *z*_(*i*+1)*x*_ at time* t*. The formulation of *p*_*z*_*ij*_*z*_(*i*+1)*x*__^*k*^(*t*) can be described as follows:

(10) $$\begin{array}{c} p_{z_{ij}z_{(i+1)x}}^{k}\left(t\right)=\{\begin{array}{cc} \frac{{\left[\tau_{\left[z_{ij}][z_{(i+1)x}\right]}\left(t\right)\right]}^{\alpha }{\left[\eta_{\left[z_{ij}][z_{(i+1)x}\right]}\left(t\right)\right]}^{\beta }}{\sum_{x,x\in \text{C}{\text{P}}_{\left(i+1\right)}}^{}{\left[\tau_{(i+1)x}\left(t\right)\right]}^{\alpha }{\left[\eta_{(i+1)x}\left(t\right)\right]}^{\beta }} & \\ z_{(i+1)x}\notin \text{tab}{\text{u}}_{k},\begin{array}{c} x\in \text{C}{\text{P}}_{\left(i+1\right)} \end{array} & \\ 0\;\;\;\text{otherwise}, & \end{array} \end{array}$$

where tabu_*k*_ represents the next node set which ant *k* cannot go to. *α* is a positive parameter, whose value represents the relative influence of pheromone trail. It shows the relative importance of ant track. The bigger the value of *α* is, the ant more inclines to choose the path which others have passed. *β* is a positive parameter, whose value represents the relative influence of heuristic information. The value of *β* is bigger, the state transition probability will be close to the greed rules. *η*_[*z*_*ij*_][*z*_(*i*+1)*x*_]_(*t*) is a heuristic function, whose value represents the expected next node (*z*_(*i*+1)*x*_) what the ant expects to choose. The value of *η*_[*z*_*ij*_][*z*_(*i*+1)*x*_]_(*t*) can be gained by (11), where *c*_(*i*+1)*x*_ represents the completion time of next node:

(11) $$\begin{array}{c} \eta_{\left[z_{ij}][z_{(i+1)x}\right]}\left(t\right)=1-\frac{c_{(i+1)x}}{\sum_{x,x\in \text{C}{\text{P}}_{\left(i+1\right)}}^{}c_{(i+1)x}}. \end{array}$$

*Pheromone Updating*. Pheromone evaporation is inevitable; meanwhile, the ants deposit pheromone in each iteration. So the pheromone value *τ*_*z*_*ij*__(*t*) is changing in each iteration. The pheromone value *τ*_*z*_*ij*__(*t*) can be updated as following equations:

(12) τ[zij][z(i+1)x](t+1) =(1−ρ)τ[zij][z(i+1)x](t)+Δτ[zij][z(i+1)x](t),

(13) $$\begin{array}{c} \Delta \tau_{\left[z_{ij}][z_{(i+1)x}\right]}\left(t\right)=\sum_{k=1}^{N_{\text{ant}}}\Delta {\tau^{k}}_{\left[z_{ij}][z_{(i+1)x}\right]}, \end{array}$$

(14) Δτk[zij][z(i+1)x] ={QZkif  ant  k  travels  on⁡  edge  (zij,z(i+1)x)0otherwise,

where *ρ* (0 < *ρ* < 1) is the rate of pheromone evaporation, and 1 − *ρ* is the rate of pheromone retention. Reducing the pheromone values enables the algorithm to forget bad decisions made in previous iterations [27]. Thus, the pheromone updating can help ants to explore new area in the search space. Δ*τ*_[*z*_*ij*_][*z*_(*i*+1)*x*_]_^*k*^ represents the pheromone value deposited on edge (*z*_*ij*_, *z*_(*i*+1)*x*_) by ant *k* at *t *iteration; Δ*τ*_[*z*_*ij*_][*z*_(*i*+1)*x*_]_ is the sum of the pheromone value deposited on edge (*z*_*ij*_, *z*_(*i*+1)*x*_) by all of ants; *Q* represents pheromone strength that affects the convergence speed of the algorithm. *Z*_*k*_ represents the objective function value of ant *k* in this iteration.

The step by step details are given as shown in Algorithm 1.

### 4.3. Fit Function

Minimizing weighted completion time is objective function, and the fitness function can be obtained by the exponential transform of objective function:

(15) $$\begin{array}{c} f=a\exp\left(-b\sum_{i=1}^{n}w_{ij}c_{ij}z_{ij}\right), \end{array}$$

where *a* is a positive real number, *b* is obtained by formula (18)

(16) $$\begin{array}{c} b=\{\begin{array}{cc} N\times M\times \frac{f_{\min}}{f_{\min}+f_{\max}} & f\geq f_{\text{avg}}\\ N\times M\times \frac{f_{\max}}{f_{\min}+f_{\max}} & f\leq f_{\text{avg}}, \end{array} \end{array}$$

where *N* is evolution generation, *M* is the number of individuals in the population, *f*_ave_ is the average fitness, *f*_min⁡_ is the minimum fitness of the individual,*f*_max⁡_ is the maximum fitness of the individual, and *f* is the individual fitness.

### 4.4. Selection

The hierarchical genetic algorithm allows the population to progress from one generation into the next. The selection process is based on the fitness of the individuals, higher fitness results in more frequent selection. There are different selection rules such as the roulette wheel implementation, tournament selection, and elitism. Roulette wheel selection method is used.

Firstly, the fitness of individual *i*(*f*_*i*_) is calculated by formula (15), then the selected probability of individual *i* can be calculated by the following formula:

(17) $$\begin{array}{c} P_{i}=\frac{f_{i}}{\sum_{k=1}^{n}f_{k}}. \end{array}$$

Secondly, the cumulative probability of each chromosome is calculated by formula (20):

(18) $$\begin{array}{c} q_{i}=\sum_{i=1}^{l}P_{i}, \end{array}$$

where *l* represents the iteration times.

Final, using roulette selection method selects the individual.

### 4.5. Crossover

**Figure 3:**
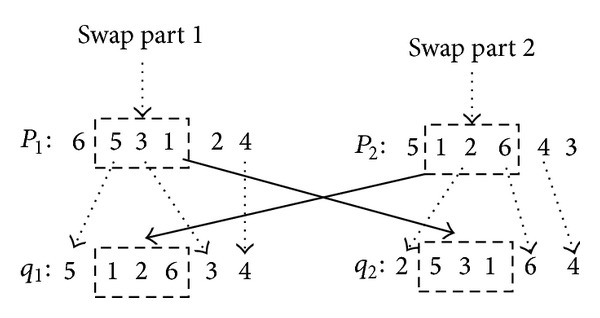
The process of crossover operator for control gene.

Some of the genetic material of two individuals are swapped (i.e., crossover operator), creating new individuals (children), who are possibly better than their parents. There are different crossover operator such as mapping crossover, different location crossover, same location crossover and leading crossover. For control gene of chromosome, partially mapping crossover method is used. The process of partially mapping crossover is that firstly, selecting two crossover points from parents' chromosome; secondly, the fragment of parents chromosome between the two crossover points is exchanged; thirdly, for the other genes, if the genes do not belong to the exchanged fragment of parents chromosome, the genes retain their value, otherwise, the value of those genes can be got using partially mapping method. For example, two parent individuals is *p*_1_ = [6 5 3 1 2 4] and *p*_2_ = [5 1 2 6 4 3], if the crossover point is 2 and 4, the offspring of individuals is *q*_1_ = [5 1 2 6 3 4] and *q*_2_ = [2 5 3 1 6 4]. The process of crossover operator is shown by Figure 3. The illegal individuals can be avoided with this method.

As the cutting scheduling problem involves process constraints, the crossover operator cannot be used for the parameter genes of chromosome. For example, the cutting pattern *P*_1_ and *P*_2_ are respectively assigned to the cutting machine *M*_5_ and *M*_1_, after some crossover operator, the cutting pattern *P*_1_ and *P*_2_ may be, respectively, assigned to the cutting machine *M*_1_ and *M*_5_, but the cutting pattern *P*_1_ cannot be cut on cutting machine *M*_1_. So some illegal individuals can be generated by using crossover operator for the parameter genes of chromosome.

An adaptive process dynamically adjusts the operator's probabilities during the process of evolving a solution [28]. In order to accelerate evolutional speed and enlarge searching scope, an adaptive crossover probability *p*_cross_ is designed:

(19) pcross={pcross×11−fmin⁡/fmax⁡if  fmin⁡fmax⁡>epcrossothers,

where *f*_ave_ is the individual's average fitness of each generation, *f*_min⁡_ is the individual's minimum fitness of each generation, *f*_max⁡_ is the individual's maximum fitness of each generation. *f*_min⁡_/*f*_max⁡_ is a positive parameter, whose value reflects the concentration of the whole generation. The bigger the value of *f*_min⁡_/*f*_max⁡_ is, this algorithm is more likely to fall into local optimal solution. If the the value of *f*_min⁡_/*f*_max⁡_ exceed a previously set threshold value *e*  (0 < *e* < 1), the individuals tend to concentration.

### 4.6. Mutation

In order to explore new areas of the search space, the mutation with introducing a variation in the population and avoid premature convergence is needed. There are two types of mutation operators: control genes mutation and parameter genes mutation. For control genes of chromosome, changing sequence variation is used. In other words, randomly two points of parent chromosome is selected, then the value of the selected point of parents chromosome is exchanged each other. For example, the parent chromosome is *p* = [6 5 3 1 2 4], selected the variation point is 2 and 5, the offspring chromosome is *p* = [6 2 3 1 5 4] after variation operator. For parameter genes of chromosome, the integer variation is used, that is, the parent parameter gene is replaced by an integer *k* (*k* ∈ C*P*_*i*_) with a certain probability.

An adaptive mutation probability *p*_muta_ is designed as follows:

(20) $$\begin{array}{c} p_{\text{muta}}=\{\begin{array}{cc} p_{\text{muta}}\times \frac{1}{1-f_{\min}/f_{\max}} & \text{if}\;\frac{f_{\min}}{f_{\max}}>r\\ p_{\text{muta}} & \text{others}. \end{array} \end{array}$$

### 4.7. Stop Criteria

In this paper, setting a maximum iteration number has been used, and the algorithm will stop when the iteration reaches the setting maximum iteration.

## 5. Computational Experiments

In this section, the proposed optimization approach is proven to be available, and the performance of the solution strategy is evaluated by describing an experiment. First of all, a real set of cutting patterns, given by the metal forming factory, is tested by this approach. Later, the test results are reported and analyzed. The date of cutting patterns is given in Table 2, and the cutting machine date has been given in Table 1 in Section 2.

In this paper, the hierarchical genetic algorithm, ant colony algorithm and ant colony—hierarchical genetic algorithm are used for this experiment. Where, the initial population size of hierarchical genetic algorithm is 100; the maximum iterations number of hierarchical genetic algorithm is 200; the threshold value *e* is 0.7; the threshold value *r* is 0.8; the initial probability of control genes crossover *p*_cross_ is 0.7, the initial probability of control genes mutation *p*_muta_ is 0.6, the initial probability of parameter genes mutation is *p*_muta_ 0.4; the information heuristic factor *α* of ant colony algorithm is 0.9; the expect heuristic factor *β* of ant colony algorithm is 6; the pheromone evaporation rate *ρ* is 0.3, the maximum iterations number of ant colony algorithm is 200, the initial population size of of ant colony algorithm is 30; the intensity of pheromone *Q* is 1000.

**Figure 4:**
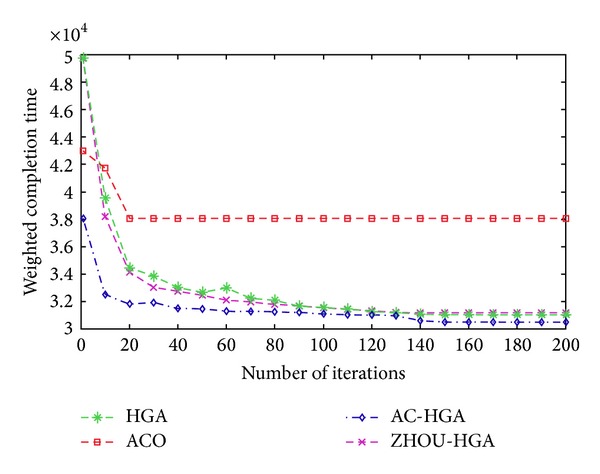
Average curve evolution.

**Figure 5:**
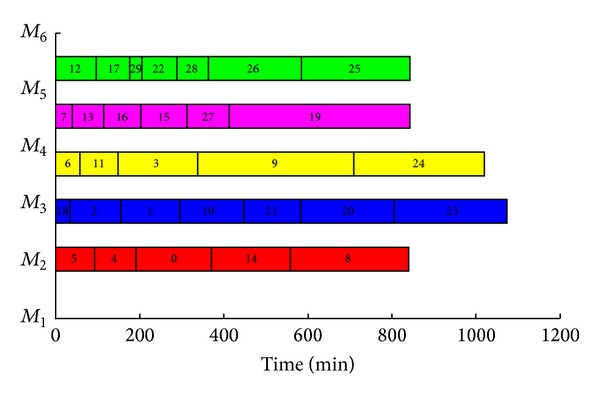
The Gantt chart of each cutting machine.

The result of the example cutting scheduling problem with process constraints used different algorithms is given by Table 3. The evolution curve of different algorithms is also shown in Figure 4. Learned from Table 3, the result of using ant colony—hierarchical genetic algorithm is best. The minimizing weighted completion time is 30510 min. The Gantt chart of each cutting machine is shown in Figure 5.

## 6. Conclusion and Deductions

This paper discusses the scheduling problem of integrated cutting stock with process constraints. The objective of minimizing weighted completion time of *n* cutting patterns for *m* nonidentical parallel machines is considered. There are three tasks contributing to solving the cutting scheduling problem. Firstly, a mathematical model for the cutting scheduling problem with process constraints is proposed. Secondly, an ant colony—hierarchical genetic algorithm is designed to solve the mathematical model. In the proposed technique, a set of initial solution is generated by ant colony algorithm, and then an optimum solution is obtained by using selection, crossover, and mutation of the hierarchical genetic algorithm. Where, the control genes are used to determine the sequence of the cutting pattern, and the parameter genes are employed to identify the cutting pattern assigned on cutting machine. Finally, computational experiment is performed to evaluate the performance of the proposed algorithm. It is verified that the proposed method on cutting scheduling problem with process constraints is effective. In addition, as Figure 4 shown, there reaches a conclusion that the proposed method overweighs other methods. The result of cutting scheduling problem is given by using Gantt chart.

The cutting scheduling plays an important role in sheet cutting process. The existing literatures give a deep research on cutting-stock problem and the combined cutting-stock and lot-sizing problem, but the study of the cutting scheduling problem has been ignored. To the best of our knowledge, the present paper is an effort to consider the cutting scheduling problem with process constraints for the first time, which involve *n* cutting patterns for *m* nonidentical parallel machines with process constraints. It belongs to scheduling problem for nonidentical parallel machines with process constraint and sequence-independent setups.

To solve this problem better, an improving hierarchical genetic algorithm is applied. A new encoding mode, that is, the natural number encoding couple with binary encoding, is adopted in this algorithm. In addition, to speed up convergence rates and resolve local convergence issues, a kind of adaptive crossover probability and mutation probability is used in this algorithm. Our approach for solving cutting scheduling problem has been applied in practical production process and has been accepted by sheet cutting manufacturers of china. This approach is novel and gives a different perspective for the manufacturing management of sheet cutting.

In this paper, the goal is focus on the minimizing weighted completion time. To achieve the target for solving the cutting scheduling problem, the minimum tardiness or other objectives can be considered. However, some products which needs sheet cutting do not merely includes cutting process (such as structural parts, after cutting process, its need to be bended, or other machined). In the future work, we will focus on the cutting scheduling problem with multi-processes.

## Figures and Tables

**Algorithm 1:**
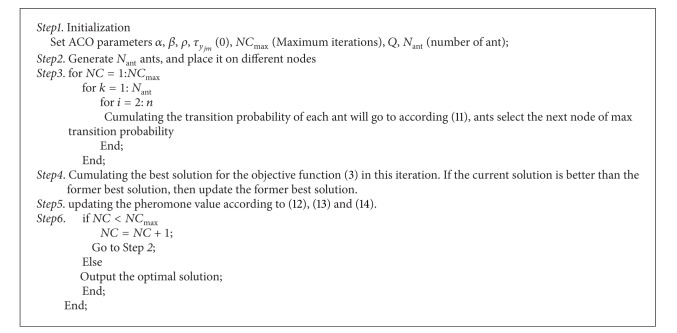


**Table 1:** The capacity of different cutting machines.

Machine no.	Cutting machine type	The range of sheet thickness can be processed.	Cutting speed
*M*_1_	Laser cutting machine	0.15 mm~6 mm carbon steel	10000 mm/min
*M*_2_	Plasma cutting machine A	1 mm~15 mm carbon steel	1000 mm/min
*M*_3_	Plasma cutting machine B	1 mm~25 mm carbon steel	800 mm/min
*M*_4_	Flame cutting machine A	6 mm~200 mm carbon steel	350 mm/min
*M*_5_	Flame cutting machine B	6 mm~200 mm carbon steel	450 mm/min
*M*_6_	Flame cutting machine C	6 mm~200 mm carbon steel	500 mm/min

**Table 2:** The data of cutting patterns

Cutting pattern	Weight	Cutting length	Number of parts	Number of punch	Material	Thickness	Available cutting machine
*P*_1_	5	43256	29	34	*Q*235	8	*M*_2_, *M*_3_, *M*_4_, *M*_5_, *M*_6_
*P*_2_	2	16656	13	16	*Q*235	8	*M*_2_, *M*_3_, *M*_4_, *M*_5_, *M*_6_
*P*_3_	4	12533	26	36	*Q*235	8	*M*_2_, *M*_3_, *M*_4_, *M*_5_, *M*_6_
*P*_4_	3	28768	22	22	*Q*235	10	*M*_2_, *M*_3_, *M*_4_, *M*_5_, *M*_6_
*P*_5_	5	11465	17	20	*Q*235	10	*M*_2_, *M*_3_, *M*_4_, *M*_5_, *M*_6_
*P*_6_	4	11909	22	29	*Q*235	10	*M*_2_, *M*_3_, *M*_4_, *M*_5_, *M*_6_
*P*_7_	3	118920	37	44	*Q*235	10	*M*_2_, *M*_3_, *M*_4_, *M*_5_, *M*_6_
*P*_8_	1	3763	5	8	*Q*235	10	*M*_2_, *M*_3_, *M*_4_, *M*_5_, *M*_6_
*P*_9_	5	32729	34	38	*Q*235	12	*M*_2_, *M*_3_, *M*_4_, *M*_5_, *M*_6_
*P*_10_	3	17464	21	29	*Q*235	12	*M*_2_, *M*_3_, *M*_4_, *M*_5_, *M*_6_
*P*_11_	4	18671	27	35	*Q*235	12	*M*_2_, *M*_3_, *M*_4_, *M*_5_, *M*_6_
*P*_12_	4	72579	33	39	*Q*345	15	*M*_2_, *M*_3_, *M*_4_, *M*_5_, *M*_6_
*P*_13_	5	58832	38	48	*Q*345	15	*M*_2_, *M*_3_, *M*_4_, *M*_5_, *M*_6_
*P*_14_	4	218320	31	43	*Q*345	15	*M*_2_, *M*_3_, *M*_4_, *M*_5_, *M*_6_
*P*_15_	1	13640	11	19	*Q*345	15	*M*_2_, *M*_3_, *M*_4_, *M*_5_, *M*_6_
*P*_16_	3	11393	19	27	*Q*345	15	*M*_2_, *M*_3_, *M*_4_, *M*_5_, *M*_6_
*P*_17_	5	106680	38	50	*Q*345	15	*M*_2_, *M*_3_, *M*_4_, *M*_5_, *M*_6_
*P*_18_	5	5928	8	15	*Q*345	20	*M*_3_, *M*_4_, *M*_5_, *M*_6_
*P*_19_	3	9564	9	13	*Q*345	20	*M*_3_, *M*_4_, *M*_5_, *M*_6_
*P*_20_	5	83235	17	23	*Q*345	20	*M*_3_, *M*_4_, *M*_5_, *M*_6_
*P*_21_	1	29441	7	17	*Q*345	20	*M*_3_, *M*_4_, *M*_5_, *M*_6_
*P*_22_	2	133620	27	36	*Q*345	20	*M*_3_, *M*_4_, *M*_5_, *M*_6_
*P*_23_	5	71432	14	18	*Q*345	24	*M*_3_, *M*_4_, *M*_5_, *M*_6_
*P*_24_	1	162360	35	42	*Q*345	24	*M*_3_, *M*_4_, *M*_5_, *M*_6_
*P*_25_	1	69178	10	16	*Q*345	24	*M*_3_, *M*_4_, *M*_5_, *M*_6_
*P*_26_	2	85800	12	16	*Q*345	24	*M*_3_, *M*_4_, *M*_5_, *M*_6_
*P*_27_	1	125400	32	39	*Q*345	30	*M*_4_, *M*_5_, *M*_6_
*P*_28_	4	159370	26	34	*Q*345	30	*M*_4_, *M*_5_, *M*_6_
*P*_29_	2	144100	34	46	*Q*345	32	*M*_4_, *M*_5_, *M*_6_
*P*_30_	1	57115	12	19	*Q*345	32	*M*_4_, *M*_5_, *M*_6_

**Table 3:** The comparison of different algorithms.

Optimization method	Optimum solution	Average value (20)	Iterative times
ACO	38063	38063	200
HGA	31063	31078	200
AC-HGA	30510	30537	200
ZHOU-HGA [25]	31198	32007	200
